# *Trichoderma viride *cellulase induces resistance to the antibiotic pore-forming peptide alamethicin associated with changes in the plasma membrane lipid composition of tobacco BY-2 cells

**DOI:** 10.1186/1471-2229-10-274

**Published:** 2010-12-14

**Authors:** Mari Aidemark, Henrik Tjellström, Anna Stina Sandelius, Henrik Stålbrand, Erik Andreasson, Allan G Rasmusson, Susanne Widell

**Affiliations:** 1 Department of Biology, Lund University, Sölvegatan 35, SE-223 62 LUND, Sweden; 2 Plant Biology Department, Michigan State University, East Lansing, 48824, MI, USA; 3 Department of Plant and Environmental Sciences, Göteborg University, P.O. Box 461, SE-405 30 Göteborg, Sweden; 4 Department of Biochemistry, P.O. Box 124, SE-221 00 Lund, Sweden; 5 Department of Plant Protection Biology, Swedish Agricultural University, P.O. Box 102, SE-230 53 Alnarp, Sweden

## Abstract

**Background:**

Alamethicin is a membrane-active peptide isolated from the beneficial root-colonising fungus *Trichoderma viride*. This peptide can insert into membranes to form voltage-dependent pores. We have previously shown that alamethicin efficiently permeabilises the plasma membrane, mitochondria and plastids of cultured plant cells. In the present investigation, tobacco cells (*Nicotiana tabacum *L. cv Bright Yellow-2) were pre-treated with elicitors of defence responses to study whether this would affect permeabilisation.

**Results:**

Oxygen consumption experiments showed that added cellulase, already upon a limited cell wall digestion, induced a cellular resistance to alamethicin permeabilisation. This effect could not be elicited by xylanase or bacterial elicitors such as flg22 or elf18. The induction of alamethicin resistance was independent of novel protein synthesis. Also, the permeabilisation was unaffected by the membrane-depolarising agent FCCP. As judged by lipid analyses, isolated plasma membranes from cellulase-pretreated tobacco cells contained less negatively charged phospholipids (PS and PI), yet higher ratios of membrane lipid fatty acid to sterol and to protein, as compared to control membranes.

**Conclusion:**

We suggest that altered membrane lipid composition as induced by cellulase activity may render the cells resistant to alamethicin. This induced resistance could reflect a natural process where the plant cells alter their sensitivity to membrane pore-forming agents secreted by *Trichoderma spp*. to attack other microorganisms, and thus adding to the beneficial effect that *Trichoderma *has for plant root growth. Furthermore, our data extends previous reports on artificial membranes on the importance of lipid packing and charge for alamethicin permeabilisation to *in vivo *conditions.

## Background

Plants possess defence systems against microorganisms that are evolutionary conserved, as well as more specialised systems that are only found in certain taxa. The conserved defence system is often referred to as the innate immunity system and this has been overcome by many successful pathogens [1] via production of pore-forming toxins or injection of pathogen effectors through pores in the plant plasma membrane [2]. Many pathogenic actions can be counteracted by recognition events via receptors coded by resistance genes [3]. The triggered defence responses are elicited by signals, either derived from the invading organism (pathogen-associated or microbe-associated molecular patterns; PAMP and MAMP, respectively) or from the plant (host-associated molecular patterns). One response is to induce programmed cell death at the attacked site, elicited by *hrp *gene products such as the pore-forming peptide harpin [4] or by products of *avr *genes like AvrD [5]. Depending on the type of threat, the final outcome can also be production of antimicrobial agents, strengthening of physical barriers such as the cell wall or detoxification of pathogen toxin [6].

Some non-pathogenic organisms *e.g*., the fungi *Trichoderma spp*. that live in the rhizosphere are antagonistic to plant pathogens, yet induce defence responses in the plants [7-10]. Several elicitors for plant defence have been identified in *Trichoderma *species and strains *e.g*., xylanase [11], hydrophobin-like proteins [12], secondary metabolites [10,13] and peptaibols [14]. The peptaibol alamethicin elicits emission of volatiles [15], induces long distance signalling [16] and also apoptosis-like death of plant cells [17]. Besides being elicitors to defence responses, the channel-forming peptaibols secreted by *Trichoderma *also kill pathogenic fungi and bacteria around the root [18,19]. Therefore, a diverse array of antimicrobial peptides isolated from *Trichoderma *and other organisms have been explored for use in plant disease control [20]. The properties of alamethicin from *T. viride *have been most intensely investigated [21,22]. This peptide is hydrophobic, 20 residues long and rich in α-amino isobutyric acid [23]. Its hydrophobic nature allows it to be inserted into biological membranes and form unspecific ion channels (pores) traversing the membranes. After insertion, the cells leak and eventually become lysed [24]. In artificial systems, pores will only form through membranes that have a transmembrane potential, and only when the alamethicin is applied from the net positive compartment [21,25]. Such a polarity of permeabilisation has been shown also *in vivo *in tobacco cells, where the plasma membrane (negative transmembrane potential) but not the tonoplast (positive transmembrane potential) was permeabilised by alamethicin added to cells [26]. With artificial membranes, several peptide molecules may oligomerise in membrane to form a barrel-stave complex with up to approximately 10 Å pore size, if a sufficient concentration of alamethicin is present [27]. Besides a negative transmembrane potential, pore formation also depends on peptide concentration, lipid/peptide ratio, lipid species, pH and ionic concentration [25,28-30]. For example, varying the size of the headgroups in artificial phospholipid bilayers affected the concentration of alamethicin needed for permeabilisation [31].

Recently, we have shown that alamethicin forms pores in plant plasma membranes, the inner mitochondrial membrane and the plastid inner envelope [26,28,32]. In short-term experiments (10 min exposure to alamethicin) with tobacco BY-2 and *Arabidopsis *col-0 cell cultures, metabolic processes could be investigated *in situ*, *i.e*., when the crowdedness of the cytosol/organelle was left intact. The permeabilisation of isolated mitochondria was nearly instantaneous [28] whereas it took several min for the plasma membrane to be completely permeabilised [26,32] suggesting that either the cell wall constituted a barrier for diffusion for alamethicin, or membrane composition affected the rate of permeabilisation.

The fact that alamethicin permeabilises plant membranes might appear incomprehensible with a beneficial role of *T. viride*. However, our experiments were done with sterile cells that had not been exposed to *T. viride*, and the situation is far from the soil situation where fungus and plant grow together and influence each other. The objective of the present investigation was to investigate if different treatments of plant cells known to induce defence responses, affect subsequent permeabilisation by alamethicin. Upon alamethicin permeabilisation the cells become depleted of respiratory metabolites. Effects of different agents on permeabilisation can therefore be monitored as differences in respiration rate decline upon alamethicin addition. Since alamethicin pore formation depends on several parameters (*e.g*., transmembrane potential and lipid composition), these properties were analysed using uncouplers and isolated plasma membranes, respectively. We here show that cellulase, unlike several other agents, made the cells resistant to subsequent alamethicin permeabilisation. Furthermore, plasma membranes isolated from cellulase-treated cells were altered in their lipid composition. We suggest that the cellulase activity induces a defence system in the plant cells and that this makes them resistant to alamethicin. These results thus provide a possible explanation for how *Trichoderma ssp*. can have beneficial effects without damaging the plants.

## Results

### Tobacco cells treated with cell wall degrading enzymes become resistant to alamethicin

**Figure 1:**
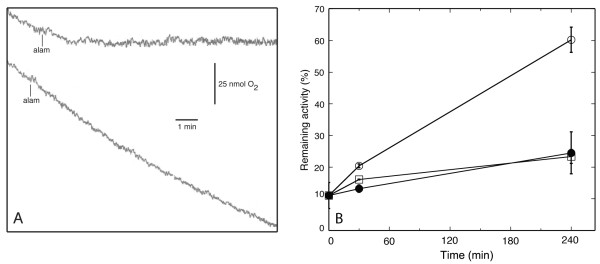
**The effect of alamethicin on oxygen consumption of tobacco cells pretreated with cellulase and macerozyme (CM)**. (A) Respiration in Control cells (upper trace) and cells treated for 4 h with CM (lower trace). Alam, addition of alamethicin. (B) Alamethicin resistance after different incubation times in Control medium and CM, respectively. Resistance was measured as per cent of respiration rate remaining after 10 min incubation with 20 µg ml^-1 ^alamethicin compared to the initial rate. Squares are control samples, open circles are CM-treated samples, and filled circles are samples treated with boiled CM. Values represent the mean of three biological replicates and the error bars denote SE.

Cultured tobacco cells respire with a relatively constant rate as long as they are intact, which can be monitored using an oxygen electrode (Figure 1). Upon alamethicin addition, the respiration rate declines over 10 min, during which time the cells become depleted for substrates and coenzymes [26]. When the cells were pre-exposed to cell wall degrading enzymes (cellulase and macerozyme in 0.35 M mannitol, pH 5.0; CM) for 4 h they retained 60% of the respiration after alamethicin addition compared to approximately 20% for cells incubated in Control medium (0.35 M mannitol, pH 5.0). At this stage of limited wall degradation, cells still retained their shape, but cell separation had begun. No visual changes in intracellular morphology (*e.g*. vacuolisation) between these cells were observed (Additional file 1). The concentrations of cellulase and macerozyme in the CM mixture (1% and 0.1%, respectively) are the ones commonly used in the isolation of protoplasts, but higher temperatures than used here are needed for a removal of the cell wall to occur within 4 h. After the same incubation at higher temperatures the resulting protoplasts, fully devoid of cell wall, were also found to be alamethicin-resistant (results not shown). However, since additional cellular changes are associated with protoplast formation, we did not further investigate protoplasts. Inactivating the enzymes by boiling before CM incubation prevented the elicitation of resistance (Figure 1), suggesting that the enzyme-induced activity on the cell wall was needed for the response. Also, lowering the incubation time in the CM medium to an initial 20 min followed by washing and incubation for 220 min with Control medium alone resulted in similar resistance compared to the full 4 h enzyme treatment (Table 1).

**Table 1:** Alamethicin resistance of tobacco cells treated with CM for different times before transfer to Control medium.

Incubation in CM-medium (min)	Postincubation in Control medium (min)	Resistance (%)
240	0	71 ± 1.4
20	220	84 ± 5.6
0	240	25 ± 3.0

Resistance was measured as per cent of respiration rate remaining after 10 min incubation with 20 µg ml^-1 ^alamethicin compared to the initial rate. Average of two independent experiments are shown with error bars representing SD.

**Figure 2:**
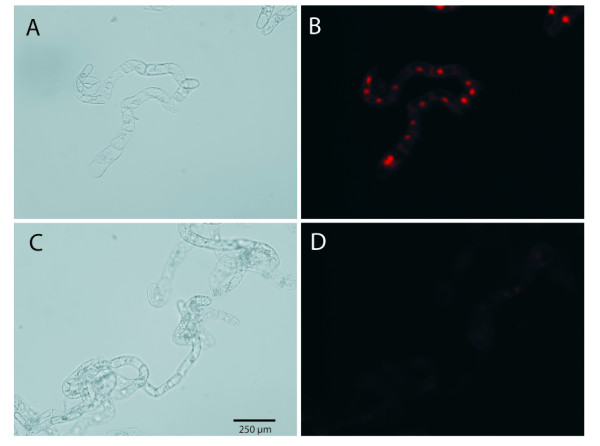
**Propidium iodide staining of alamethicin-treated tobacco cells**. Bright field (A) and (C) and fluorescent (B) and (D) images are shown for cells after incubation with 20 µg ml^-1 ^alamethicin for 10 min. Before addition of alamethicin, cells were pretreated with either Control medium for 4 h (A, B) or CM medium for 20 min followed by 220 min with Control medium (C, D). The bar is valid for all images.

The DNA stain propidium iodide cannot pass the plasma membrane of intact cells and can therefore be used as direct indicator of alamethicin permeabilisation [32]. Control cells showed strong fluorescence of the nucleus after incubation with alamethicin and propidium iodide (Figure 2), while only a faint signal could be observed in cells treated for 20 min with CM medium, followed by 220 min with Control medium (Figure 2). No staining was observed in the absence of alamethicin in any cells.

**Figure 3:**
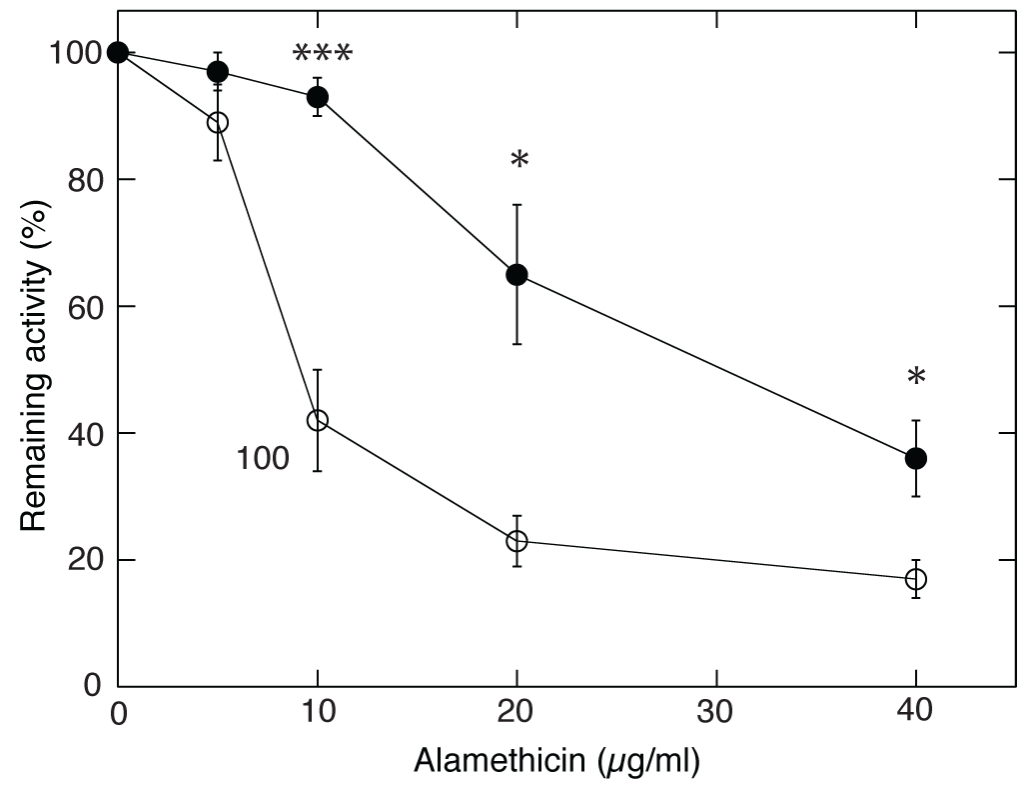
**Remaining respiration in control and CM-treated tobacco cells after adding different concentrations of alamethicin**. Open circles, control cells; filled circles CM-treated cells. Resistance was measured as per cent of respiration rate remaining after 10 min incubation with 20 µg ml^-1 ^alamethicin compared to the initial rate. Each data point represents the mean of four biological replicates and the error bars represent SE. Significant differences (Student's t-test) between CM cells and control are denoted with * for p < 0.05 and *** for p < 0.001.

In the above experiments, 20 µg ml^-1 ^alamethicin was used to permeabilise the cells. We compared the concentration dependence of alamethicin permeabilisation between control cells and CM-treated cells, and significant differences were observed over an extended range (Figure 3). At 40 µg ml^-1 ^alamethicin, also CM-treated cells became permeabilised, though not to the same extent as control cells (Figure 3). The concentration dependency showed a sigmoid pattern with both control and CM cells. Approximately three times the concentration of alamethicin was needed with CM-treated cells compared to control cells to yield a 50% permeabilisation, *i.e*., 30 µg ml^-1 ^for CM cells compared to less than 10 µg ml^-1 ^for control cells (Figure 3).

### Alamethicin resistance of tobacco cells is mainly due to the effect of cellulase

In the initial experiments, cells were treated with a combination of cellulase and macerozyme in mannitol (CM). To determine whether both enzymes were needed for the elicitation of alamethicin resistance we also treated cells with each of the enzymes separately. It was found that cellulase was more important than macerozyme for the development of resistance, since cellulase alone induced almost the same level of resistance as the CM treatment did (Table 2). As little as 0.05% cellulase, one twentieth of the concentration normally used in a protoplast preparation mix, gave an increased resistance to alamethicin relative to the control. With 0.1% macerozyme alone (the concentration normally used in a protoplastation mix) a limited resistance developed (Table 2).

**Table 2:** Alamethicin resistance of tobacco cells treated with different concentrations of cellulase and macerozyme.

Cellulase (%)	Macerozyme (%)	Resistance (%)
0	0	27.1 ± 4.4
1	0.1	74.2 ± 5.6
1	0	64.0 ± 4.2
0.05	0	43.8 ± 3.9
0	0.1	37.3 ± 5.3
0	0.05	29.2 ± 4.0

Resistance was measured as per cent of respiration rate remaining after 10 min incubation with 20 µg ml^-1 ^alamethicin compared to the initial rate. Average of two independent experiments are shown with error bars representing SD.

**Figure 4:**
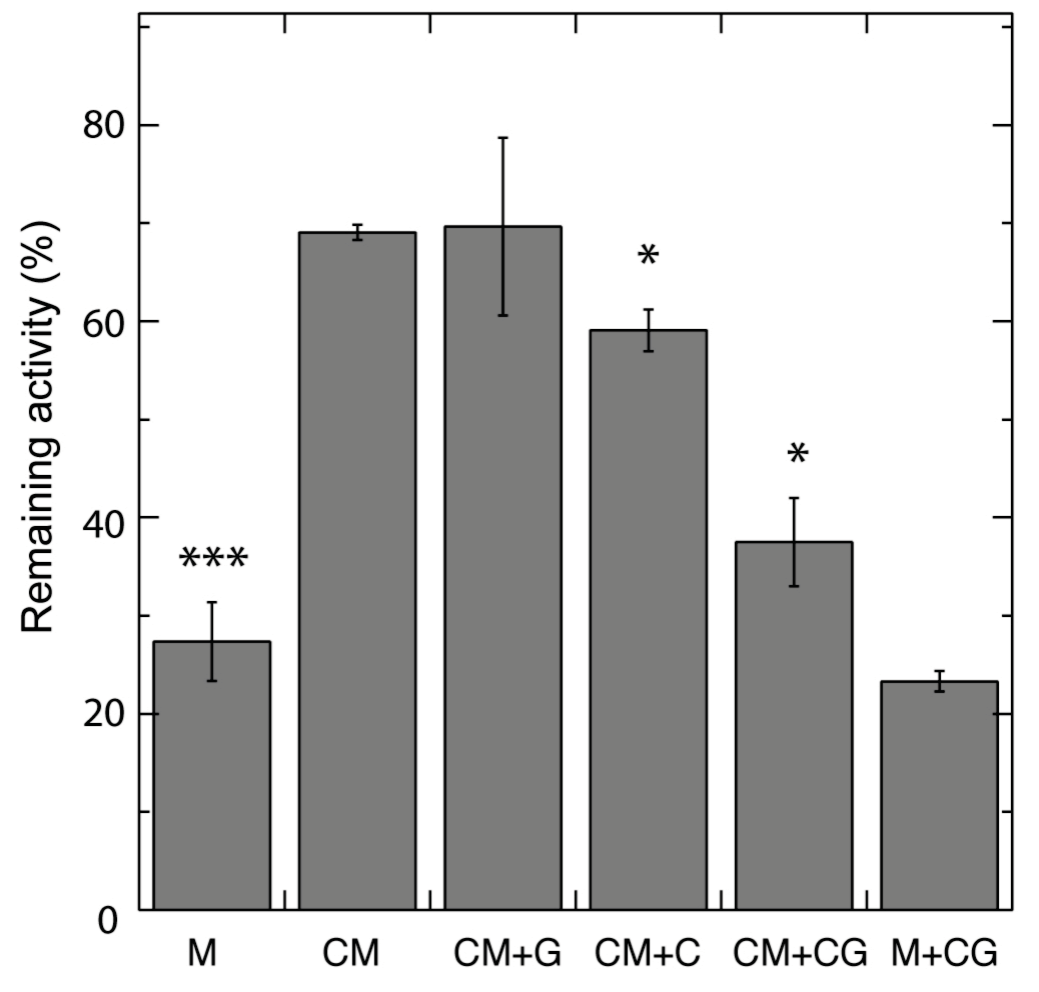
**Effect of inhibition of cellulase activity on the induction of alamethicin resistance of tobacco cells**. Resistance was measured as per cent of respiration rate remaining after 10 min incubation with 20 µg ml^-1 ^alamethicin compared to the initial rate. Samples were pre-incubated with combinations of 1% cellulase, 0.1% macerozyme, 0.1 M glucose, and 0.1 M cellobiose in 0.35 M mannitol for 20 min followed by 220 min with control medium only. Where glucose or cellobiose was included, the concentration of mannitol in the control medium was reduced to give a similar molarity. M, control cells, CM, CM-treated cells, G, glucose, C, cellobiose. Data shown are averages of two biological replicates and error bars represent SD. Student's t-test was performed relative to the CM sample with * denoting p< 0.05 and *** denoting p < 0.001.

Cellulase from *T. viride *contains a mixture of endoglucanases, exoglucanases and β-glucosidases [33]. Both the endo- and exoglucanases of the *T. viride *cellulase are product-inhibited by cellobiose, while the β-glucosidase is product-inhibited by glucose [34,35]. Because of this, we tested to inhibit the induction of alamethicin resistance by adding glucose and cellobiose to the incubation mixture. The concentrations used were considerably higher than reported K_i _values for endo/exo-glucanases and β-glucosidase, and thus significant inhibition of the enzymes can be assumed [34-37]. Addition of cellobiose alone lowered the alamethicin resistance induced by enzyme treatment of cells (Figure 4). This effect increased when 0.1 M glucose was included with the cellobiose to inhibit β-glucosidase degradation of the cellobiose. Glucose by itself had no effect on the alamethicin resistance of CM treated samples (Figure 4). The observation that cellulase inhibition reduced the resistance to alamethicin shows that the cellulase activity is important for the elicitation of alamethicin resistance.

The cellulase preparations used are relatively crude and effects seen could potentially be batch-dependent. However, similar degrees of resistance could be induced using a second cellulase batch from the same supplier (Yakult Honsha) and one from Serva (Table 3). Both these cellulases are from *T. viride*. In contrast, no resistance could be induced by Celluclast, a cellulase mixture that is isolated from *T. reesei *and used to degrade cellulose industrially (Table 3). After establishing that endoglucanases or exoglucanases in the cellulase mixture were the main source of the elicited alamethicin resistance we tested additional enzymes for elicitation potential. No resistance was obtained after incubating cells 4 h with *T. reesei *endoglucanase TrCel7Bcor or *T. reesei *endomannanase TrMan5A (Table 3).

**Table 3:** Alamethicin resistance of cells treated with different cell wall degrading enzymes or enzyme modules.

Enzyme	Source species	Resistance (%)
Cellulase (Yakult)	*T. viride*	76 ± 12
Cellulase (Serva)	*T. viride*	75 ± 9
Celluclast	*T. reesei*	18 ± 5
TrCel7Bcor module	*T. reesei*	22 ± 3
TrMan5A module	*T. reesei*	19 ± 2

Resistance was measured as per cent of respiration rate remaining after 10 min incubation with 20 µg ml^-1 ^alamethicin compared to the initial rate. Average of two independent experiments are shown with error bars representing SD.

### Several common plant elicitors did not induce alamethicin resistance

**Figure 5:**
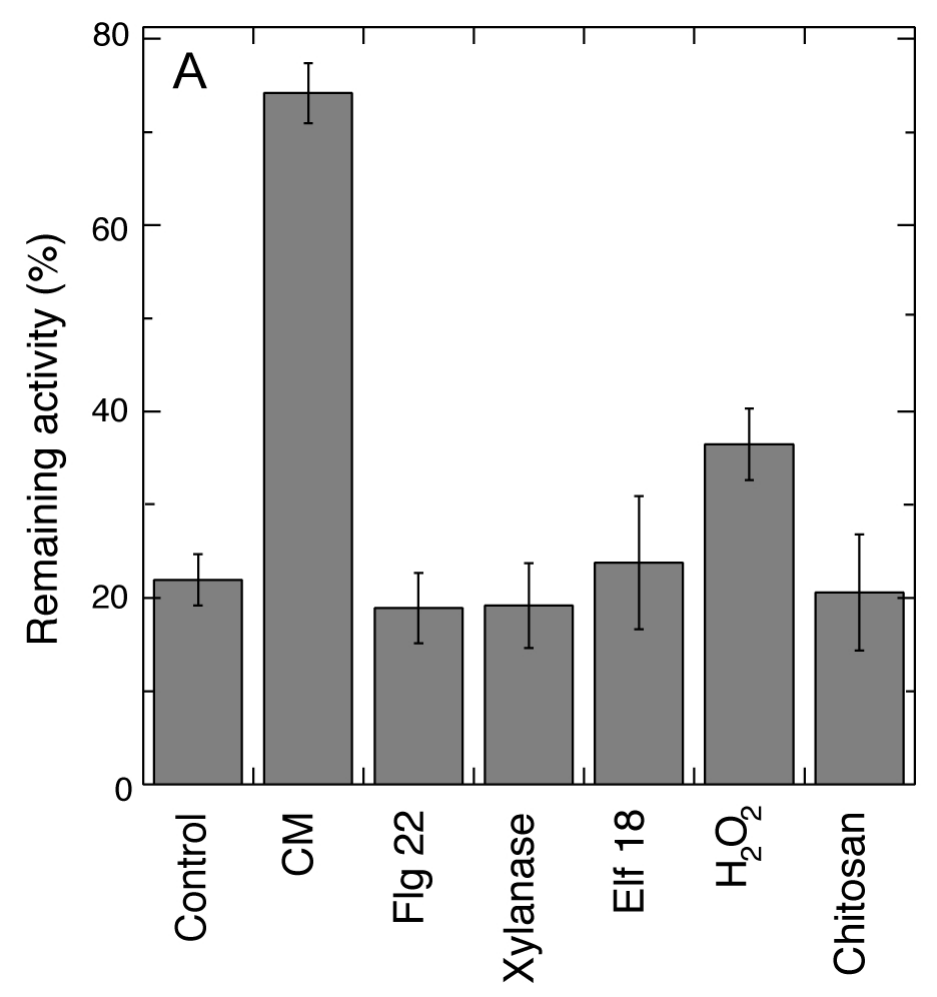
**Resistance to alamethicin after preincubation of tobacco cells with known plant defence elicitors**. Resistance was measured as per cent of respiration rate remaining after 10 min incubation with 20 µg ml^-1 ^alamethicin compared to the initial rate. Data points are averages of three to five measurements and error bars represents SE.

To find out how general the alamethicin resistance response was, other elicitors of defence responses in plants were investigated. No resistance to alamethicin was induced by 4 h incubation with xylanase, elf18, flg22 or chitosan (Figure 5). As positive controls for the treatments with xylanase, elf 18 and flg 22 treatments, MAP kinase activation was monitored after these treatments (results not shown). A low level of alamethicin resistance could be seen after treatment with 1 mM H_2_O_2 _(Figure 5). However, adding catalase during CM treatment did not prevent the induction of alamethicin resistance (Additional file 2). None of the elicitors examined gave an alamethicin resistance in the vicinity of that attained after CM treatment (Figure 4). In addition, cells were incubated with a low level (1 µg ml^-1^) of alamethicin during 4 h to find out if alamethicin by itself could elicit a resistance to further exposure. However, no difference in remaining respiration after regular alamethicin permeabilisation was evident (24 ± 7% in alamethicin-treated cells as compared to 22 ± 7% for the control cells).

### Alamethicin resistance develops independently of protein synthesis and membrane depolarisation

**Figure 6:**
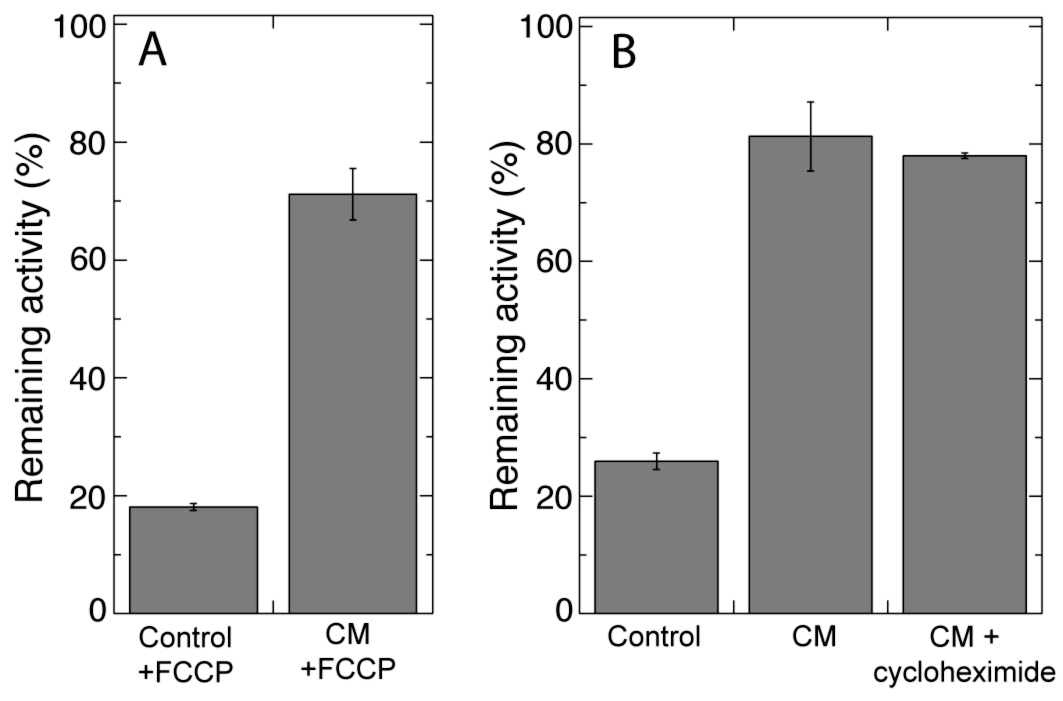
**The effect of the uncoupler FCCP (A) and protein synthesis inhibitor cycloheximide (B) on the CM-induced alamethicin resistance of tobacco cells**. Resistance was measured as per cent of respiration rate remaining after 10 min incubation with 20 µg ml^-1 ^alamethicin compared to the initial rate. Average of two independent experiments are shown with error bars representing SD. FCCP was added just before alamethicin addition, whereas cycloheximide was added before CM treatment (as described in Methods). The respiration increased 1.6 ± 0.1 and 1.7 ± 0.4 times in control and CM-treated cells, respectively, by the addition of FCCP, showing that respiration in the cell cultures became equally uncoupled from ATP synthesis.

It could not be excluded that the CM-treatment induced a plasma membrane depolarisation sufficient to slow down the permeabilisation process or change the amount of alamethicin needed. Therefore, we tested the effect on alamethicin permeabilisation by the protonophore FCCP, which depolarises the transmembrane potential to the diffusion potential in maize roots [38] and abolishes adenylate control of respiration in tobacco cells [39]. As expected, FCCP activated respiration in both control and CM-treated cells, but alamethicin-permeabilisation of control cells was unaffected by the FCCP (Figure 6). Consistently, CM-treated cells were similarly resistant to alamethicin in the presence of FCCP (Figure 6A) as in its absence (Figure 6B, Control).

We then investigated whether the alamethicin resistance of the tobacco cell cultures involved *de novo *protein synthesis. The presence of the protein synthesis inhibitor cycloheximide prior to and during incubation with CM did not affect the magnitude of alamethicin resistance (Figure 6B). This indicates that posttranslational changes are sufficient for induction of alamethicin resistance.

### CM treatment results in distinct plasma membrane lipid profile alterations

**Figure 7:**
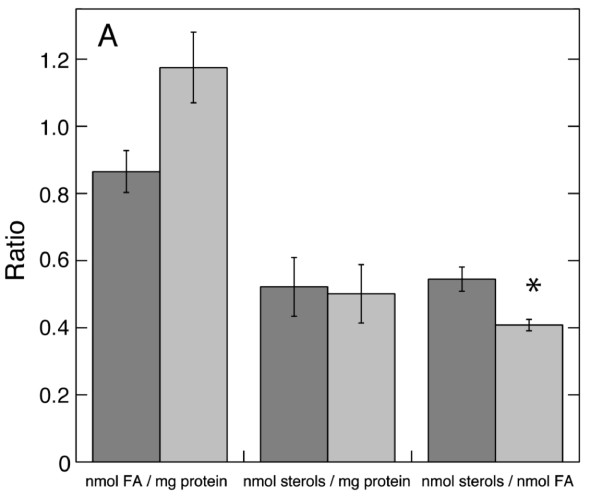
**Protein, fatty acid and sterol ratios in plasma membranes isolated from control and CM-treated cells**. Dark grey bars, control cells; light grey bars, CM-treated cells. Values used are averages of two plasma membrane preparations and error bars denote SD.

As mentioned, alamethicin permeabilisation depends on membrane lipid composition in artificial systems [21]. This suggests that the resistance induced by cellulase seen here with tobacco cells, could be caused by changes in the membrane lipids. Plasma membranes were therefore isolated from control cells and CM-treated cells (Additional file 2). The total amount of membrane lipid fatty acids per protein increased more than 30% in plasma membranes of CM-treated cells compared to control (Figure 7). The sterol/protein ratio did not change, which means that the ratio of sterol to fatty acid decreased. The main sterols found in the plasma membrane of both control and enzyme-treated cells were campesterol, stigmasterol and β-sitosterol (Additional file 3). No changes in the relative amounts of the individual sterols were observed (Additional file 3). The ratio of acetylated sterol glycosides compared to free sterol, decreased from 0.39 ± 0.03 in control to 0.32 ± 0.03 for CM-treated samples.

**Figure 8:**
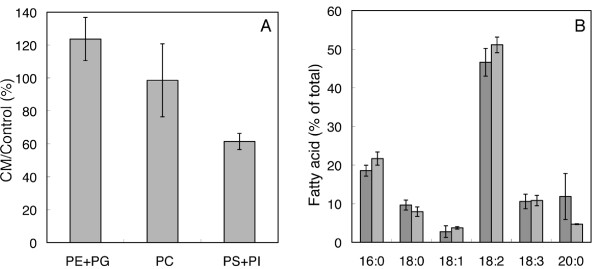
**Phospholipid analysis of tobacco cell plasma membranes**. **(**A) Percents of different phospholipids of plasma membranes from CM-treated cells relative to control cells. The CM/Control ratio for PS+PI was significantly different from that for PE+PG (p < 0.05). (B) Fatty acid composition of plasma membranes isolated from control and CM-treated cells. Dark grey bars, control cells; light grey bars, CM-treated cells. Values used are averages of two plasma membrane preparations and error bars denote SD.

Differences were found in the amounts of plasma membrane phospholipids between control and CM-treated cells. Figure 8A shows that the most prominent change was a drastic lowering in phosphatidylserine and phosphatidylinositol (PS+PI) after CM-treatment. In contrast, we observed an increase in phosphatidylethanolamine (PE) detected together with phosphatidylglycerol (PG), but PE constituting at least 95% of the sum (results not shown). The responses to CM treatment for PS+PI and PE+PG were significantly different (p < 0.05). PS and PI are negatively charged phospholipids (at neutral pHs) as are phosphatidic acid (PA) and PG, whereas PE and phosphatidylcholine (PC) are zwitterionic and net uncharged molecules. Similar changes were not seen in the microsomal fractions, from which the plasma membranes were isolated (results not shown). The most common membrane lipid fatty acid in the plasma membrane of both control and CM-treated cells was 18:2 (linoleic acid) followed by 16:0 (palmitic acid; Figure 8B). No large changes in fatty acid species were induced by CM treatment except possibly for a CM-induced drop in 20:0 (arachidic acid). A small decrease in saturation was found in the CM-treated cells, *i.e*., the ratio between saturated and unsaturated fatty acid corresponded to 0.63 ± 0.05 in control membranes compared to 0.55 ± 0.04% in membranes from CM-treated cells.

## Discussion

Biocontrol fungi such as *T. viride *are known to induce systemic resistance, ISR, and prime their host plants to become more resistant to future attack from pathogenic microorganisms [9,40]. The transcriptional changes related to ISR are usually quite modest compared to systemic acquired resistance, SAR [41]. We here found that treatment of tobacco cells with *T. viride *cellulase resulted in posttranslational changes leading to altered membrane properties and alamethicin resistance. To the best of our knowledge, the presented data are the first to show that resistance to permeabilisation by the peptaibol alamethicin can be induced in any eukaryote. Interestingly, cell wall degrading enzymes and peptaibols from *T. harzanium *synergistically prevented spore germination and hyphal growth of *Botrytis cinerea *[42]. Thus, synergies that are harmful to one system (*Trichoderma *on pathogen) can be protective in another system (*Trichoderma *on plant), which favours a successful symbiotic relation between *Trichoderma *and the plant.

The alamethicin resistance observed was mainly elicited by the enzymatic activity of *T. viride *cellulase. This is strongly indicated by the reduction in elicited resistance by heat inactivation and by the presence of the cellulase inhibitor cellobiose. Further, the effect of inhibitors excludes the possibility of alamethicin resistance being elicited by any of the small known contaminants of most cellulase extracts. Shortening the enzyme incubation to 20 min followed by a post-incubation in Control medium alone (until the same total of 4 h had passed) did not reduce the alamethicin resistance induced. This indicates that the cellulase elicits the resistance during the first part of the incubation and that no further stimulus is required, but that it takes a certain time for the response to develop in the plant cell. After these treatments, no visual changes could be observed by light microscopy, indicating that only a limited cell wall digestion had taken place. Interestingly, the observed resistance displays some specificity for *T. viride *cellulases since the effect was neither seen upon incubation with a cellulase mixture from *T. reesei *nor by hemicellulases of the same fungus (Figure 5 Table 3). The presence of a cellulose-binding module (frequently carried by cellulases) did not induce resistance, consistent with the inactivation and inhibition studies showing that an active enzyme was needed (Figure 1 Figure 4).

It could be argued that the resistance observed here is a part of a general defence response to cell wall degradation, intended to increase the robustness of the plasma membrane in anticipation of a fungal or bacterial attack reaching through the cell wall. It has earlier been reported that cellulase treatment can evoke defence responses, *e.g*., increases in the stress-related phytoalexin capsidiol [43,44] as well as the production of volatile compounds [45,46]. Xylanase, which can degrade the xylan of the cell wall hemicelluloses represents a threat to cell integrity similar to that posed by cellulase [47,48]. However, in contrast to the eliciting effect of cellulase in our experiments, xylanase does not need to be enzymatically active to elicit defence responses in tobacco [49]. Also, the difference in mode of elicitation is consistent with the inability of xylanase to elicit alamethicin resistance.

If alamethicin resistance were part of a general response to pathogen attack it would be reasonable to assume that many common plant elicitors mediated a similar response. The acetylated chitin derivate chitosan is able to elicit a large range of plant defensive responses, including HR, SAR, oxidative burst and callose deposition [50], yet we could not detect a significant difference in alamethicin resistance. Similarly, with the PAMPs flg 22 [51] and elf 18 [52], no elicitation of alamethicin resistance could be observed, despite their ability to trigger innate immunity. Finally, adding catalase to cells during CM did not prevent the elicitation of resistance (Additional file 2). This indicates that the somewhat increased resistance observed after H_2_O_2 _incubation is not due to H_2_O_2 _being a putative intermediate in the cellulase-initiated signalling cascade. Instead, the presence of H_2_O_2 _can lead to rapid cross-linking of the cell wall proteins [53]. The decrease in permeability of the cell wall after such cross-linking may be the reason for the moderate alamethicin resistance after H_2_O_2 _incubation (Figure 5). In any case, this resistance at the cell wall level cannot explain the cellulase-induced alamethicin resistance, since also protoplasts devoid of cell wall were resistant to alamethicin.

Rather, the alamethicin resistance could be compared to classical R-gene-induced resistance in the sense that both might counteract pore formation activities of successful pathogens and beneficial microorganisms. Instead of manipulating the consequences of pores by deactivating the pathogen effectors that are transported through them, as is characteristic to R gene-mediated resistance, the alamethicin resistance decreases the possibility for pores to be formed.

Analyses conducted with artificial lipid bilayers have suggested that alamethicin needs to be delivered from the compartment with the net positive electric potential in order to be inserted and form pores in membranes [21]. Experimental data on biological systems are in line with this, *i.e*., the vacuole (which has a positive transmembrane potential) in tobacco cells was left intact under conditions when other membranes were permeabilised [26]. Upon cellulase treatment, the transmembrane potential of *Medicago sativa *root hairs was depolarised to ca -50 mV [54], *i.e*., to what probably would be the diffusion potential [55]. However, for the resistance development described here, transmembrane potential changes could be ruled out as important since no effect was obtained by the protonophore FCCP (Figure 6), an agent shown to depolarise the transmembrane potential in roots to the diffusion potential [38]. Also, protein synthesis was not needed for the process (Figure 6), showing that the resistance depended on modifications performed by pre-existing enzymes or structures. Cell wall modifications induced by the action of *T. viride *cellulase may result in both chemical and mechanical signals reaching the plant cell. Cellodextrins (β-1,4 glucose oligomers), *i.e*., the predominant breakdown products of cellulose, induced pathogen responses in *Vitis vinifera *[56]. On the other hand, homologues of prokaryotic and eukaryotic mechanosensitive channels were recently identified in *A. thaliana *[57], and an existence of mechanosensing signalling also in plants has recently been suggested [58]. However, the lack of effect by xylanase in our experiments (Figure 5) and the quite small effect induced by macerozyme (Table 2) shows that if the signal is mechanical, it cannot operate simply through the degradation of classical matrix polysaccharides.

Peptide-induced pore formation depends on membrane lipid species and lipid/peptide ratio [31]. We found that the sterol to membrane lipid fatty acid ratio (Figure 7), the fraction of PS+PI (Figure 8) and the acyl group 20:0 decreased as a consequence of enzyme treatment. Our analyses were performed with cells that still were indistinguishable from untreated cells with regard to shape (Additional file 1), but when substantial alamethicin resistance could be detected. Therefore, the changes in lipid composition seen probably reflect the defence induced against *T. viride*, whereas the degradative changes often associated with complete protoplastation [59-61] are kept at a minimum. This also agrees with that strains of *Staphylococcus aureus*, *Enterococcus faecalis *and *Bacillus cereus *with a five-fold increased resistance to alamethicin permeabilisation (IC_50 _of 2-5.5 µg ml^-1 ^alamethicin in sensitive and 9.5 to 29 µg ml^-1 ^in resistant strains, respectively), showed altered membrane lipid composition as well as lower alamethicin association to vesicles prepared from membrane extracts [62].

The CM-induced changes in phospholipids and their corresponding fatty acids (Figure 7 Figure 8), suggest that the physical properties of the plasma membrane were altered, possibly sufficient to affect alamethicin insertion and pore formation. This agrees with that the conductance through pores made by the antimicrobial cationic peptide gaegurin 4 was larger in planar bilayers made of PE, PC and PS (80:10:10) compared to membranes composed of only PE and PC (80:20) [63]. A role of sterols with respect to alamethicin channel activity was shown with artificial membranes, *i.e*., the presence of cholesterol increased the duration of the alamethicin pore in its open state, indicating a more efficient use of created pores, while the critical concentration of alamethicin needed for pore formation increased [64,65]. Oligomerisation and pore formation by *Vibrio cholerae *cytolysin also depended on the presence of cholesterol [66]. With gaegurin 4 [63], inclusion of cholesterol in planar lipid membranes acted opposite to PS, *i.e*., it prevented channel formation. This deviates from the association of increased alamethicin resistance to decreased sterol levels (relative to fatty acids) observed with tobacco cells (Figure 7). However, the hydrophobic alamethicin forms pores that traverse the membrane through its hydrophobic part, whereas cationic peptides such as gaegurin 4 form pores in the membrane where peptide and membrane lipid headgroups are exposed to the inner of the pore [67]. Besides, the presence of proteins in biological membranes adds another degree of complexity, making direct comparisons between peptide types difficult.

Large differences in lipid composition were used in the above investigations of alamethicin pore formation with artificial membranes. This might speak against direct comparisons with the smaller differences found for the tobacco plasma membrane here, also since the artificial membranes do not contain proteins as do biological membranes. However, effector-induced changes in membrane phospholipids and sterols of similar magnitudes as we found with tobacco lead to changes in membrane stability with isolated plasma membranes from oat roots [68] and *S. cerevisiae *[69] as seen by changes in transversal bilayer diffusion.

Another important property of especially the phospholipids is their charge, with PC and PE being uncharged and PA, PI, PS and PG being negatively charged. The charges of the lipid head groups and the membrane proteins will cause a local surface charge which will affect the attraction of ions to approach the membrane, and also modulate the spacing of lipids. In our experiments, we found that CM treatment resulted in lower PM-associated PS+PI and higher PE (+PG) compared to control cells (Figure 8A). Even though the surface charges depend also on *e.g*., proteins and the phospholipid distribution between the respective plasma membrane leaflets, the results suggest that overall surface charge of the plasma membrane may be lower in CM-treated cells compared to control cells. With artificial membranes, lower surface charge result in less alamethicin inserted [70].

## Conclusions

*T. viride *cellulase treatment made tobacco cells resistant to permeabilisation by alamethicin. Several changes in the lipid composition of plasma membrane were found, suggesting a change in membrane properties. It is conceivable that the defence response elicited by *T. viride *cellulase makes the tobacco plasma membranes resistant to alamethicin by acting on membrane properties that are needed for alamethicin insertion. In nature, plant roots are likely to encounter cellulase and alamethicin at the same time, as they are both secreted by *T. viride*. Plant cells should therefore be more sensitive at the site of first encounter during the time needed for resistance induction. However, this is not lethal, and at later stages, when a signal from the partially degraded cell wall (chemical or mechanical) have led to altered membrane properties, the plant root will have built up its resistance to alamethicin. This renders the plant root insensitive to alamethicin at concentrations that might inhibit or kill nearby microbes. The shift seen here in sensitivity to alamethicin (Figure 3) is fully in accordance with such an explanation. These findings therefore provide a model of how a beneficial microorganism can protect its symbiotic plant counterpart from pore forming molecules that it secretes to attack pathogens in the surroundings.

## Methods

### Plant material

*Nicotiana tabacum *BY-2 cells were grown on a rotary shaker at 125 rpm in constant darkness at 24°C, and subcultured every seven days as described [26]. The cells were harvested for experiments on the fourth day after subculture, during the exponential growth phase (300 - 450 mg fresh weight cells per ml medium).

### Treatments of BY-2 cells for oxygen electrode measurements and microscopy

Unless otherwise denoted, tobacco BY-2 cells were incubated for 4 h in a Control medium (0.35 M mannitol, pH 5.0) or CM medium, *i.e*., Control medium supplemented with enzymes (1% cellulase "Onozuka" RS (Yakult Honsha co., Ltd., Japan, if not otherwise stated) and 0.1% macerozyme (Yakult Honsha co., Ltd., Japan). In some experiments, the concentrations of cellulase and macerozyme were varied, and treatments were also made where the cellulase and or macerozyme was inactivated by boiling prior to addition. In other cases, cells were incubated in CM medium for 20 min and then pelleted and transferred to Control medium and incubated for another 220 min. Other treatments were: either 0.1 µg ml^-1 ^alamethicin, 100 µg ml^-1 ^xylanase from *T. viride*, 1 µM elf18 (SKEKFERTKPHVNVGTIS; Caslo Laboratory ApS, Denmark), 1 µM flg22 (QRLSTGSRINSAKDDAAGLQIA; Caslo Laboratory ApS, Denmark), 1 mM H_2_O_2_, 10 µg ml^-1 ^chitosan, 0.3 U ml^-1 ^Celluclast 1.5 L (a mixture of *Trichoderma reesei *cellulases and other plant cell wall degradative enzymes from Novozymes, Denmark) [71], 0.3 U ml^-1 ^TrCelB endoglucanase catalytic module [72], and 0.3 U ml^-1 ^TrMann5A endomannanase (carrying a cellulose-binding module [73]), all in Control medium. Combinations of 0.1 M cellobiose, 0.1 M glucose and mannitol to a total concentration of 0.35 M were added in experiments where the inhibition of cellulase was tested. Catalase was used to a final concentration of 192 U ml^-1^. This concentration is sufficient to inhibit H_2_O_2_-mediated apoplastic peroxidase cycles [26,74]. In one experiment 80 µM cycloheximide was included with the enzyme treatment, as well as 1 h prior to enzyme addition. This concentration is sufficient to inhibit inducible processes in tobacco cell suspensions [75]. In another experiment, 4 µM FCCP was added just before alamethicin addition. All treatments were performed at room temperature on a rotary shaker at 70 rpm.

### Oxygen electrode measurements

After treatments, the BY-2 cells were diluted in a measuring medium (20 mM HEPES, 60 mM MES, 300 mM mannitol, 1 mM MgCl_2 _and 1 mM EGTA, pH 7.5) to 40 mg (FW) ml^-1 ^(*i.e*., ca 10 times dilution) and oxygen consumption was measured using a 1 ml Clark Oxygen Electrode (Rank Brothers, UK). After initial measurements of cellular respiration, alamethicin (Sigma-Aldrich, Germany) was added from a stock solution (20 mg ml^-1 ^in 60% ethanol) and respiration was measured for an additional 10 min. Unless otherwise stated, a concentration of 20 µg ml^-1 ^of alamethicin was used. Resistance against permeabilisation was determined as the ratio between the slope 10 min after alamethicin addition and the initial slope (see Figure 1).

### Microscopy

BY-2 cells were treated with Control medium or CM medium for 3 h (Additional file 1), or with Control medium for 4 h respectively with CM medium for 20 min followed by 220 min with Control medium (Figure 2). Before incubation with dyes, cells were diluted to 40 mg (FW) ml^-1 ^(*i.e*., ca 10 times dilution) in measuring medium (see above). For propidium iodide staining, cells were incubated with 20 µg ml^-1 ^of alamethicin for 10 min and 1.5 µM propidium iodide (Invitrogen, Sweden) was added during the last 5 min of the alamethicin incubation.

Fluorescence microscopy was performed using a G-2A-filter (excitation at 510-560 nm, emission above 590 nm) in a Nikon-Optiphot-2 microscope (Nikon Corporation, Japan). As a reference, a bright field transmission microscopy picture was taken.

Confocal microscopy images were collected using a Zeiss LSM 510 (Zeiss, Germany).

### Plasma membrane purification

Membrane fractions were prepared from cell cultures treated with Control or CM media. The alamethicin resistance of the CM-treated cells was measured regularly using oxygen electrode respiration measurements (see above) and cells were harvested for fractionation when the alamethicin resistance was above 60%.

Cell cultures (ca 50 g per treatment) were suspended in extraction buffer (50 mM MOPS/KOH, pH 7.5, 5 mM EDTA, 330 mM sucrose, 5 mM ascorbic acid, 3 mM DTT, 0.6% (w/v) polyvinyl polypyrrolidone) and homogenized using a mixer fitted with razorblades (Braun). Extracts were filtered through a 150 µm net and centrifuged at 7,200 × g for 15 min at 4°C. The supernatants were centrifuged at 40,000 × g for 1 h at 4°C to pellet the microsomal fraction (MF). Plasma membranes (PM) and intracellular membranes (ICM) were purified from the microsomal fraction by partitioning in an aqueous polymer two-phase system [76,77]. A phase system of the following composition was used: 6.0% (w/w) Dextran T 500, 6.0% (w/w) polyethylene glycol 4000, 330 mM sucrose, 5 mM potassium phosphate (pH 7.8) and 2 mM KCl. After three partitioning steps, the fractions (PM, ICM and MF) were diluted in 250 mM mannitol, 10 mM HEPES/KOH, pH 7.5) and pelleted by centrifugation at 100,000 × g for 1 h at 4°C. Samples were resuspended in the same medium and were stored at -80 °C until use.

### Assays

The degree of purification of plasma membranes from microsomal fractions was established by comparing callose synthesis (GSII) and cytochrome *c *oxidase activity in plasma membrane and intracellular membrane fractions to that of the original microsomal fraction. Callose synthesis and cytochrome c oxidase activity was measured according to [78] and [79] respectively. Protein was determined according to Bearden [80]. To ensure that the membrane fractions obtained were of similar purity, markers for plasma membrane and mitochondria were analysed with these membrane fractions. The enrichment of callose synthase activity (plasma membrane marker) and depletion of cytochrome *c *oxidase activity (marker for the mitochondrial inner membrane) in the respective plasma membrane fraction were relatively similar (Additional file 4) showing that they were useful for comparative studies. The enrichments obtained agree well with earlier obtained data on plasma membrane purification [76,77]. MAP kinase activity was measured according to [81].

### Lipid analyses

Lipids were extracted according to Sommarin and Sandelius [82] and fractionated into neutral lipids, glycolipids and phospholipids by solid phase extraction (SPE) as described [83]. For quantification of sterols and phospholipids, internal standards were added to the lipid extracts before SPE fractionation. Sterols were analyzed after conversion to trimethylsilyl (TMS)-ethers by gas liquid chromatography (GLC) using the same setup as in described [83]. β-cholestanol and di17:0-phosphatidylcholine were used as internal standards for sterol and phospholipids, respectively. Glycolipids were analyzed by high pressure liquid chromatography (HPLC) equipped with a light scattering detector as previously described [83] and quantified using standard curves of authentic lipid standards. Fatty acid methyl esters (FAME) were produced by base catalysis of sodium-methoxide in methanol [84] and quantified on a GLC, as previously described [83]. Diheptadecanoylphosphatidylcholine was used as internal standard. Thin layer chromatography (TLC) was performed using Si60 TLC plates (VWR International, Germany) and lipids were identified by co-chromatography with authentic lipid standards (Sigma-Aldrich, USA). TLC plates were developed in CHCl_3_:MeOH:acetic acid:water (85:15:10:3.5) and the lipids were visualized by charring [85] or dichlorofluorescein treatment [86] Phospholipid proportions were quantified by densitometry using Syngene Bio imaging system (UK) and accompanying software.

## Abbreviations

CM: cellulase and macerozyme; FCCP: carbonylcyanide 4(-triflouromethoxy)phenylhydrazone; PA: phosphatidic acid; PE: phospatidylethanolamine; PG: phosphatidylglycerol; PI: phosphatidylinositol; PS: phosphatidylserine.

## Authors' contributions

SW, AR and MA conceived the study and planned the majority of the experiments. MA conducted all the experiments. HT and ASS took part in the lipid analyses and the interpretation of the results from these, HS with the experiments with glucanases and EA with the elicitors. SW and MA wrote the manuscript with substantial contribution also from AGR. All authors read, commented and approved the manuscript.

## Supplementary Material

Additional file 1 **Confocal transmission images of tobacco cells**. Images are taken after 3 h of treatment with Control medium (A), or 20 min with CM medium followed by 160 min with Control medium (B). (C, D) Magnified squared sections of A and B, respectively. The bar denotes 50 µm and is valid also for B. Click here for file

Additional file 2 **Effect of preincubation of tobacco cells with catalase on resistance to alamethicin**. Resistance was measured as per cent of respiration rate remaining after 10 min incubation with 20 µg ml^-1 ^alamethicin compared to the initial rate. Click here for file

Additional file 3 **Sterol analysis of tobacco cell plasma membranes isolated from control and CM-treated cells**. Click here for file

Additional file 4 **Membrane marker analysis of fractions from control and CM-treated cells**. Click here for file
