# Supplementary figures and images for: Trichoderma viride cellulase induces resistance to the antibiotic pore-forming peptide alamethicin associated with changes in the plasma membrane lipid composition of tobacco BY-2 cells
Source: BMC Plant Biol. 2010 Dec 14;10:274. doi: 10.1186/1471-2229-10-274 (PMC3017840; doi:10.1186/1471-2229-10-274)

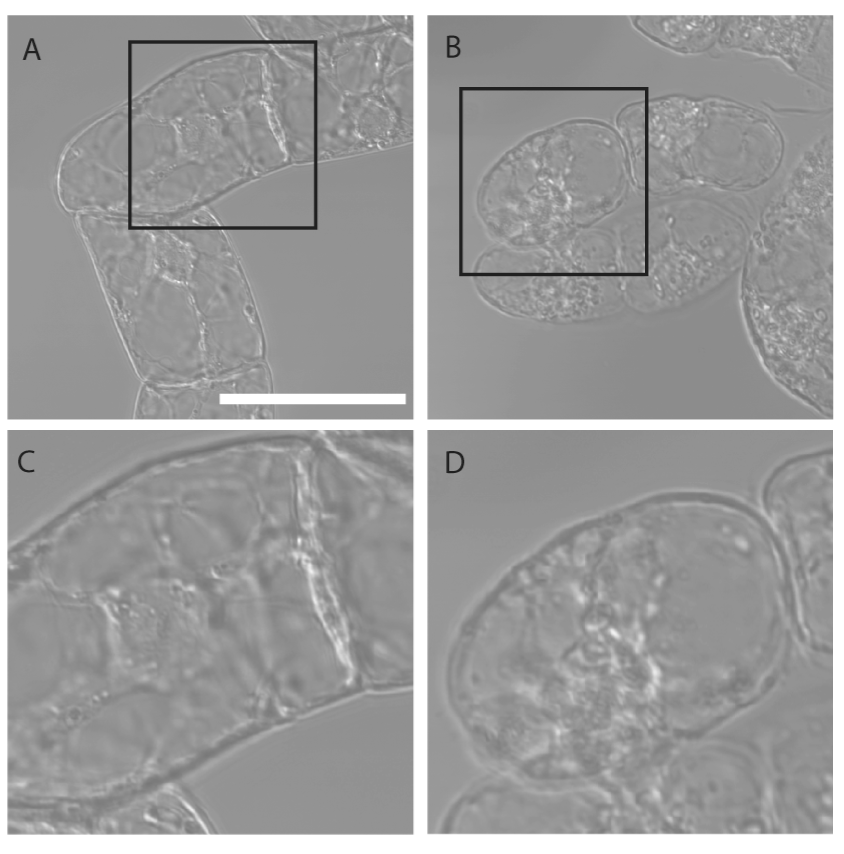

Supplement: Additional file 1 — Confocal transmission images of tobacco cells. Images are taken after 3 h of treatment with Control medium (A), or 20 min with CM medium followed by 160 min with Control medium (B). (C, D) Magnified squared sections of A and B, respectively. The bar denotes 50 µm and is valid also for B. [file 1471-2229-10-274-S1.TIFF]

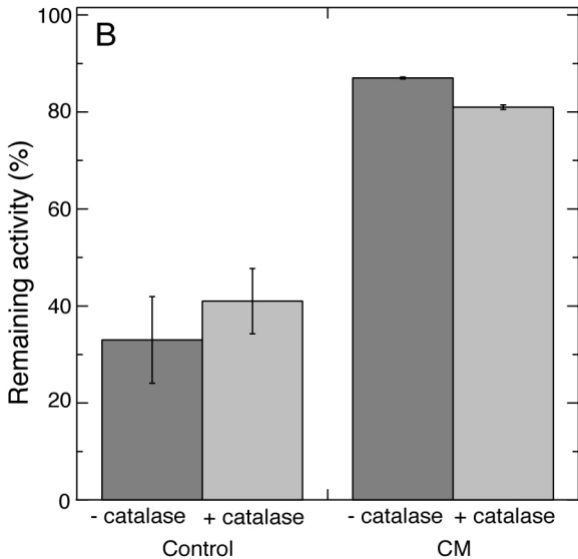

Supplement: Additional file 2 — Effect of preincubation of tobacco cells with catalase on resistance to alamethicin. Resistance was measured as per cent of respiration rate remaining after 10 min incubation with 20 µg ml-1 alamethicin compared to the initial rate. [file 1471-2229-10-274-S2.PDF]

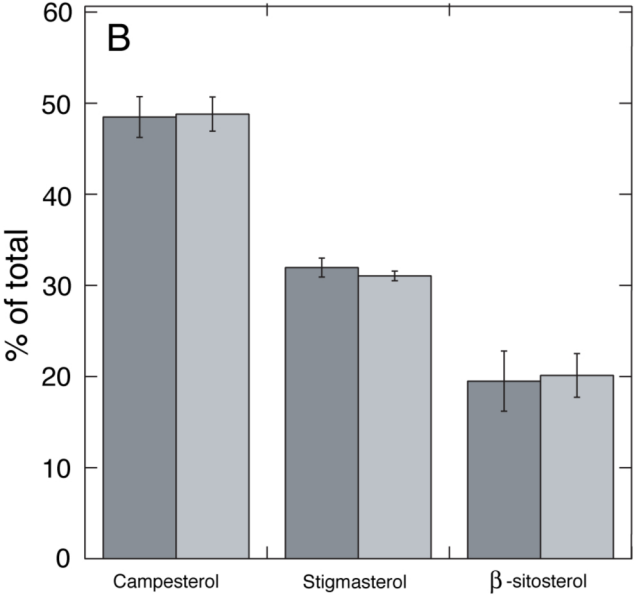

Supplement: Additional file 3 — Sterol analysis of tobacco cell plasma membranes isolated from control and CM-treated cells. [file 1471-2229-10-274-S3.PDF]
