# Supplementary material for: Trichoderma viride cellulase induces resistance to the antibiotic pore-forming peptide alamethicin associated with changes in the plasma membrane lipid composition of tobacco BY-2 cells
Source: BMC Plant Biol. 2010 Dec 14;10:274. doi: 10.1186/1471-2229-10-274 (PMC3017840; doi:10.1186/1471-2229-10-274)
Supplement: Additional file 4 — Membrane marker analysis of fractions from control and CM-treated cells. [file 1471-2229-10-274-S4.DOCX]

**Additional file 4**

**Purification of plasma membranes from Control and CM-treated cells.**

A. Specific activities

|  | Glucan synthase II | |  | Cytochrome *c* oxidase | |  |
| --- | --- | --- | --- | --- | --- | --- |
|  | nmol min^-1^ mg^-1^ | Enrichment | | nmol min^-1^ mg^-1^ | Enrichment | |
| Control cells |  |  | |  |  | |
| MF | 62 ± 30 | 1 | | 710 ± 240 | 1 | |
| PM | 640 ± 100 | 11 ± 3.7 | | 30 ± 8 | 0.04 ± 0.00 | |
| ICM | 22 ± 11 | 0.35 ± 0.02 | | 580 ± 230 | 0.81 ± 0.05 | |
| CM-treated cells |  |  | |  |  | |
| MF | 84 ± 19 | 1 | | 1400 ± 640 | 1 | |
| PM | 490 ± 70 | 5.9 ± 2.1 | | 110 ± 110 | 0.06 ± 0.05 | |
| ICM | 12 ± 12 | 0.16 ± 0.18 | | 1100 ± 460 | 0.79 ± 0.03 | |

B. Total activities

|  | Glucan synthase II | |  | Cytochrome *c* oxidase | |  |
| --- | --- | --- | --- | --- | --- | --- |
|  | µmol min^-1^ | Recovery (%) | | µmol min^-1^ | Recovery (%) | |
| Control |  |  | |  |  | |
| MF | 0.77 ± 0.08 | 100 | | 9.1 ± 0.08 | 100 | |
| PM | 0.46 ± 0.01 | 60 ± 8 | | 0.02 ± 0.0 | 0.2 ± 0.0 | |
| ICM | 0.12 ± 0.05 | 15 ± 9 | | 3.3 ± 1.9 | 36 ± 20 | |
| CM-treated cells |  |  | |  |  | |
| MF | 2.5 ± 0.9 | 100 | | 39 ± 13 | 100 | |
| PM | 1.7 ± 0.5 | 67 ± 2 | | 0.28 ± 0.2 | 0.7 ± 0.3 | |
| ICM | 0.14 ± 0.06 | 6.2 ± 4 | | 17 ± 6 | 47 ± 31 | |

A microsomal fraction (MF) was used to isolate plasma membranes (PM) and intracellular membranes (ICM) by aqueous polymer two-phase partitioning from Control and CM-treated cells. Enrichment (shown in A) is the ratio of specific activity in PM and ICM to that of MF. Recovery (shown in B) is the percentage of the total activity in MF that was retained in PM and ICM, respectively. Glucan synthase II and cytochrome c oxidase were used as markers for the plasma membrane and the inner mitochondrial membrane, respectively.
